# High incidence of asymptomatic cases during an outbreak of *Plasmodium malariae* in a remote village of Malaysian Borneo

**DOI:** 10.1371/journal.pntd.0009450

**Published:** 2021-06-03

**Authors:** Nurul Athirah Naserrudin, Emira Izzati Abdul Aziz, Erdie Aljet, George Mangunji, Bumpei Tojo, Mohammad Saffree Jeffree, Richard Culleton, Kamruddin Ahmed

**Affiliations:** 1 Borneo Medical and Health Research Centre, Faculty of Medicine and Health Sciences, Universiti Malaysia Sabah, Kota Kinabalu, Malaysia; 2 Department of Community Health, Faculty of Medicine, Universiti Kebangsaan Malaysia, Kuala Lumpur, Malaysia; 3 Kota Marudu District Health Office, Ministry of Health Malaysia, Kota Marudu, Malaysia; 4 School of Tropical Medicine and Global Health, Nagasaki University, Nagasaki, Japan; 5 Community and Family Medicine Department, Faculty of Medicine and Health Sciences, Universiti Malaysia Sabah, Kota Kinabalu, Malaysia; 6 Division of Molecular Parasitology, Proteo-Science Center, Ehime University, Toon, Japan; 7 Department of Pathobiology and Medical Diagnostics, Faculty of Medicine and Health Sciences, Universiti Malaysia Sabah, Kota Kinabalu, Malaysia; London School of Hygiene and Tropical Medicine Faculty of Infectious and Tropical Diseases, UNITED KINGDOM

## Abstract

An outbreak of *Plasmodium malariae* occurred in Sonsogon Paliu village in the remote area of Ulu Bengkoka sub-district of Kota Marudu, Northern Sabah, Malaysian Borneo from July through August 2019. This was the first outbreak of malaria in this village since 2014. On 11^th^ July 2019 the Kota Kinabalu Public Health Laboratory notified the Kota Marudu District Health Office of a Polymerase Chain Reaction (PCR) positive case of *P*. *malariae*. This index case was a male from Sulawesi, Indonesia working for a logging company operating in Sonsogon Paliu. During the resulting outbreak, a total of 14 symptomatic cases were detected. All of these cases were positive by thick and thin blood smear examination, and also by PCR. During the outbreak, a mass blood survey screening was performed by light-microscopy and PCR. A total of 94 asymptomatic villagers 31 (33.0%) were PCR positive but thick and thin blood smear negative for *P*. *malariae*. Both symptomatic and asymptomatic cases received treatment at the district hospital. When symptomatic and asymptomatic cases were considered together, males (29/45. 64.5%) were infected more than females (16/45, 35.6%), the male:female ratio being 1.8:1. Adults were the predominant age group infected (22/45, 48.9%) followed by adolescents (19/45, 42.2%) and children under five years of age (4/45, 8.9%). This report illustrates that symptomatic and submicroscopic cases pose a challenge during *P*. *malariae* outbreaks and that PCR is a valuable tool for their identification. The rapid identification and control of imported malaria is crucial for the continued control of malaria in Malaysia.

## Author summary

In Malaysia, malaria caused by the human-only malaria parasites has been all but eliminated, and the major obstacles to a malaria-free status are now emerging malaria caused by zoonotic transmission of *Plasmodium knowlesi* parasites and outbreaks caused by imported parasites. One such outbreak occurred in 2019 in a village in northern Sabah. This outbreak was unusual, as it was found to be caused by *P*. *malariae*, an often-benign human malaria parasite rarely seen in Malaysia. We investigated this outbreak and found that the index case could be traced to an Indonesian migrant worker employed in the logging industry. An additional 13 people in the village developed malaria symptoms and were subsequently found to be positive for *P*. *malariae*. A further 94 inhabitants of the village without symptoms were then tested by a sensitive PCR technique, and 31 of them were also found to be positive for the parasite. These results illustrate the threat posed by asymptomatic carriage of malaria parasites, and the importance of PCR testing for outbreaks of residual malaria in elimination settings.

## Introduction

Malaysia has been identified by the World Health Organization (WHO) as one of 21 countries with the potential to eliminate human malaria by 2020 [1]. However, imported malaria poses a major challenge to Malaysia’s efforts to eliminate the disease. Among all the states of Malaysia, Sabah has the highest number of malaria cases, the majority of which are caused by the zoonotic parasite, *Plasmodium knowlesi* [1–5]. However, cases of malaria caused by human-only malaria parasite species have decreased dramatically, with only 23 cases of *Plasmodium falciparum* and eight cases of *Plasmodium vivax* recorded in Sabah 2017 [1], whereas in the same period 3,614 cases of *P*. *knowlesi* were reported [6].

*Plasmodium malariae* infection is often asymptomatic, rarely leads to severe clinical illness or death, and is typically associated with a low-grade chronic infection that can persist for extended periods of time, perhaps even decades [7,8]. The disease has been linked to nephropathy and anemia [9,10]. Although the distribution of *P*. *malariae* is rather patchy, it has been observed in all major malaria-endemic regions of the world, throughout tropical Africa, Southeast Asia, and Central and South America [9–13]. Although responsible for far fewer cases of clinical malaria than *P*. *falciparum* and *P*. *vivax* globally, it can cause long-term chronic infections and possesses the ability to recrudesce due to the persistence of subclinical parasitemia [9]. Of the malaria parasites that commonly infect humans, *P*. *malariae* remains relatively little studied, both at the biological and epidemiological levels [7,12]. Here we report an outbreak of malaria caused by *P*. *malariae* in Sonsogon Paliu village, Kota Marudu district, Sabah during July and August 2019. Our findings highlight the challenges posed by imported malaria for the achievement of malaria elimination.

## Methods

### Ethics statement

This study was approved by the Medical Ethics and Research Committee, Ministry of Health Malaysia (NMRR-20-1523-55702). The study protocol was registered under the National Medical Research Registry. Written informed consent was obtained from participants and in the case of children informed consent was obtained from guardians.

### Outbreak investigation

On the 11^th^ of July 2019, the Kota Kinabalu Public Health Laboratory (KKPHL) notified the Kota Marudu District Health Office of a *P*. *malariae* case in a village in their administrative region. KKPHL is the reference laboratory of Sabah state for malaria and other infectious diseases. Venous blood was sent to this laboratory for confirmatory diagnosis of malaria. All cases were diagnosed using thin and thick blood smear microscopy and PCR. An outbreak investigation was carried out that involved the formation of three teams for population screening, outbreak control, and entomological investigations.

Population screening for malaria parasites was performed by Mass Blood Survey (MBS) using thin and thick blood smear microscopy and PCR. The target population for this study were all villagers who resided in the study village. Villagers’ blood was withdrawn from the cubital fossa *via* venipuncture using needles and syringes. Samples were stored in sampling tubes, and transported to the laboratory in a cold-box. Thick and thin smear microscopy was carried out within 24 hours of blood collection. Staining and microscopy were conducted according to standard operating procedures provided by the Public Health Laboratory. All slides were examined by a trained microscopist. Slides were examined at 100 × magnification and the number of parasites per 200 leukocytes determined. Slides were considered negative when no parasites were observed after counting >100 microscopic fields.

An epidemic curve was plotted using the time of first reported symptoms as a start point. Using a structured questionnaire (S1 Table), face-to-face interviews were conducted with the assistance of an interpreter. The questionnaire provided information on socio-demographic background, lifestyle, preventive measures against malaria/mosquito bites, comorbidities, consumption of traditional medicine/plants for fever, symptoms, travel history, knowledge of malaria, presence of monkeys near the house, and any recent arrival of non-local people in the village.

### Entomological investigation

The entomology team analyzed the effectiveness of vector control measures. These measures include indoor residual spraying (IRS) and the distribution of Long-Lasting Insecticide Nets (LLIN). The analysis was based on the effectiveness of IRS and LLIN and to ensure that the activities followed standard procedures. Entomological surveys were conducted for three nights from 29 July to 31 July 2020. Both indoor and outdoor landing catches were carried out with two men sitting indoors and six outdoors from 6.00 pm until 12.00 am. All staff were given doxycycline prior to the survey. Adult mosquitoes were collected using small vial tubes [14]. All mosquitoes sampled were identified and further dissected with the ovaries examined for parity, meanwhile, the midguts and salivary glands were examined for oocysts and sporozoites. Mosquito larvae were collected by dipping collection at potential breeding sites.

### Location of *P*. *malariae* cases

A map for outbreak cases was created using R, and its graph drawing packages (ggplot2, sf, and ggspatial). Natural Earth (https://www.naturalearthdata.com) administrative area data was used for administrative boundaries. Road data was obtained from OpenStreetMap Data Extracts (https://download.geofabrik.de). For the location information of each village included in the district, records from the Kota Marudu Health Office were used. The coordinates of houses of all cases were determined *via* GPS.

### Statistical analysis

Data were collected and stored in Microsoft Excel. Statistical analysis was performed using SPSS.

## Results

Sonsogon Paliu village is situated 90km from Kota Marudu city center and villagers are of Dusun ethnicity. The village is surrounded by forest and is accessible only by four wheel-drive vehicles as there are no paved roads (S1–S3 Figs). There is no electricity or potable water supply to the village. Water is primarily provided by rainwater. Cellphone signals are scarce and only available in certain hotspots. The nearest school is an eight hour walk from the village. Fishing, farming and hunting are the main occupations of the villagers whereas some work at a logging company which performs sustainable logging in the area. Logging activities cover an area of 60,000 hectares of land and workers are recruited from locals as well as migrant workers from Indonesia and the Philippines.

Malaria control measures were last implemented in the village between 17^th^ February and 31^st^ March 2019 through the distribution of insecticide treated nets (ITNs) and insecticide repellent spray to the community. As a continuation of vector control activities each malaria case received a personal repellent following discharge from hospital.

The outbreak control team identified the index case as an Indonesian male from Sulawesi who had been working for a logging company in Sonsogon Paliu from the end of March 2019. He had no history of travel to other countries except Sulawesi, from where he had travelled at the end of February 2019.

### Descriptive results

**Fig 1:**
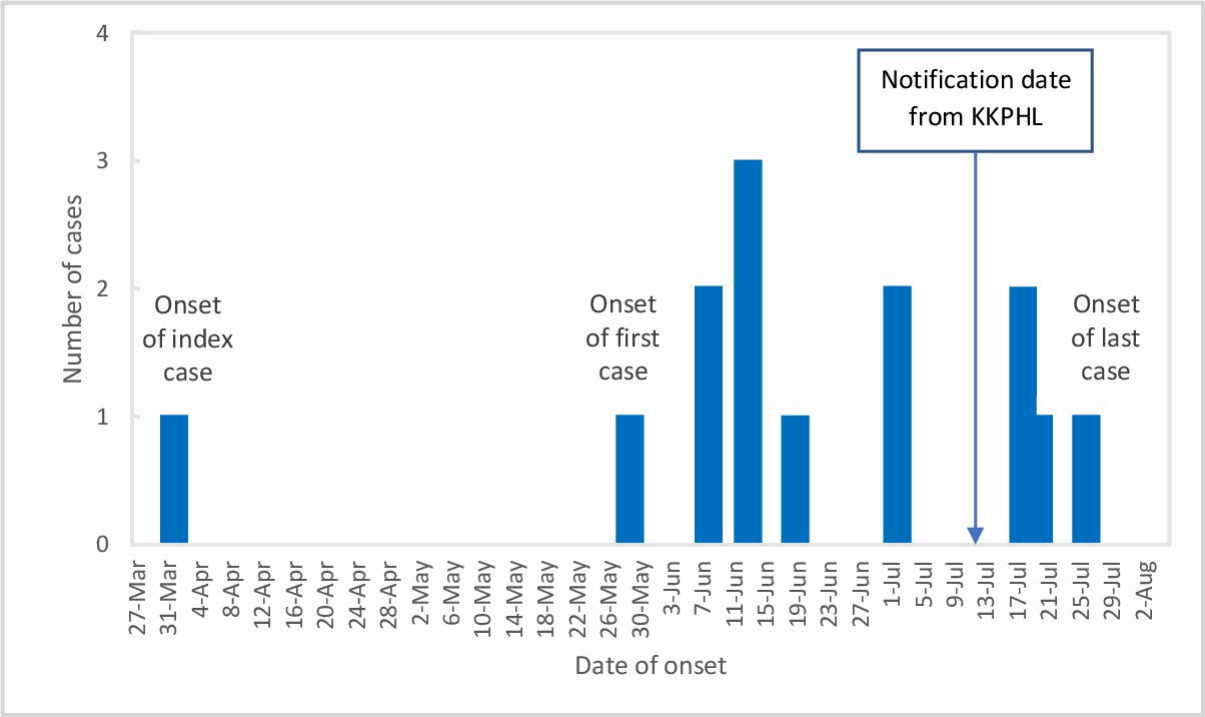
Epidemic curve. Epidemic curve of the *Plasmodium malariae* outbreak in Sonsogon Paliu village, Kota Marudu district, Sabah, Malaysia during July–August 2019. The numbers of cases are plotted against the day of occurrence of malaria.

The index case developed symptoms on 1^st^ April 2019, and an outbreak was declared on the 11^th^ of July 2019 when the district health office received a *P*. *malariae* positive PCR result. The first case-patient became symptomatic on the 31^st^ of May, and on the 12^th^ of June the peak was noted followed by a decrease with the last case-patient becoming symptomatic on the 25^th^ of July (Fig 1).

**Fig 2:**
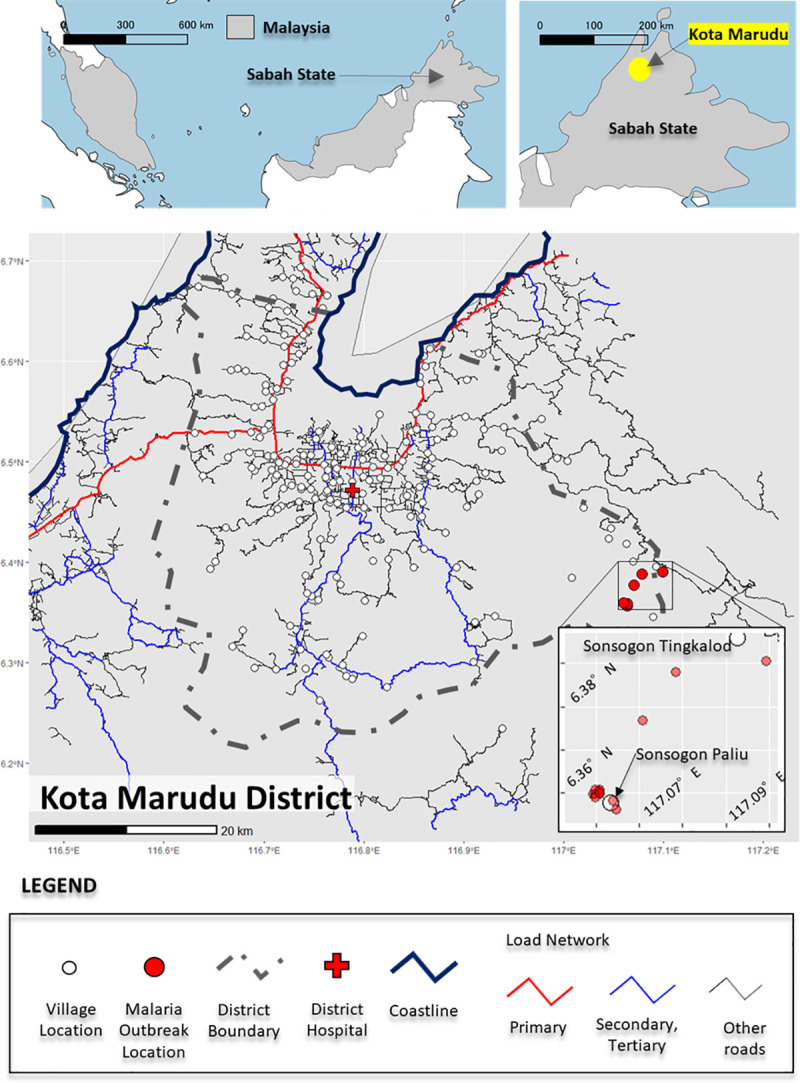
Kota Marudu district. Map of Kota Marudu district showing the location of Sonsogon Paliu village in the south-west of the district. The upper inset shows a map of Malaysia and the location of Kota Marudu district in Sabah state. There are no primary, secondary, tertiary or other road network connections to the village, and the next nearest village is more than 20km away. The lower inset is shows the symptomatic cases represented by red dots. Except for three cases the houses of all the symptomatic cases were very close to each other. Kota Marudu District Hospital is shown as a red-cross. Except for three cases the houses of all the symptomatic cases were very close to each other. Map was produced using QGIS Version 3.1.6 (QGIS Development Team, 2020). Source of shapefile: https://www.naturalearthdata.com/downloads/10m-cultural-vectors/.

There were 108 villagers in Sonsogon Palis with a median age of 22.5 years (range 2–93). Among them, 14 cases of symptomatic *P*. *malariae* infection were recorded, resulting in an attack rate (AR) of 12.96%. Among these, nine (64.3%) were male and five (35.7%) were females (male: female ratio, 1.8:1). Except for three cases the houses of all the symptomatic cases were very close to each other (Fig 2). The first case was a three year old girl; no members of her household were infected with *P*. *malariae*. Six households were identified with multiple members infected; two households had two cases, two had three cases, one had four cases, and one had five cases infected with *P*. *malariae*.

The median age of symptomatic *P*. *malariae* positive patients were 16.5 years (range 3–30 years). During the outbreak, the highest number of patients (7/14, 50.0%) belonged to the 5–18-year age group followed by those above 18 years (5/14, 35.7%) and less than five years (2/14, 14.3%). Thirty-one individuals were identified as asymptomatic *P*. *malariae* parasite carriers by MBS. Among these, 20 (64.5%) were males and 11 (35.5%) were females (ratio 1.8:1). The median age of these patients was 21 years (range 2–86 years). The highest number of patients (17/31, 54.8%) were over 18 years of age, followed by 5–18 year-olds (12/31, 38.7%) and those below five years (2/31, 6.5%). When symptomatic and asymptomatic cases were considered together, males (29/45. 64.5%) were more likely to be infected than females (16/45, 35.6%, male: female ratio, 1.8:1). Age distribution showed that adults were most at risk (22/45, 48.9%) followed by adolescents (19/45, 42.2%) and children under five years of age (4/45, 8.9%).

There were no deaths during this outbreak. All cases were treated orally with Riamet (artemether plus lumefantrine) at Kota Marudu District Hospital. There were no recorded adverse effects associated with drug treatment.

### Entomological survey

During 136 person-collecting hours, a total of seven mosquitoes were caught, four were identified as *Anopheles balabacensis*, two were identified as *Aedes albopictus* and one sample was *An*. *barbirostris*. Further dissection of mosquito ovaries revealed that all the mosquitoes were nulliparous. No oocysts or sporozoites were detected in the midguts and salivary glands respectively. All mosquitoes were collected from outdoor biting collection, and none were found indoors. *Anopheles barbirostris* larvae were detected in two ponds within 50 meters from the residence of one *P*. *malariae* positive case. Our survey also showed that the usage rate of ITNs and insecticide repellent spray by village households was 100%.

## Discussion

According to data retrieved from the Kota Marudu District Health Office, the last cases of infection by human-only malaria parasites in Sonsogon Paliu village occurred in 2014. These were a case of *P*. *vivax* and three cases of *P*. *falciparum*. From April 2014 onwards, no cases of malaria caused by human-only malaria parasite species were reported, however, there were 20 cases of *P*. *knowlesi* infection during this period. Therefore, when the index case was first notified it was suspected to be a case of *P*. *knowlesi* as this is the dominant species in Sabah [1–6]. *Plasmodium malariae* accounts for only 0.6–0.8% of *Plasmodium* infections in Sabah [1,2] and the PCR result confirming the index case to be infected with this species was surprising. However, *P*. *malariae* is endemic in Sulawesi, Indonesia [15,16], where the index case travelled from. Although travel history might suggest that the index case was infected in Indonesia, we cannot rule out the possibility that he was infected in Sabah.

The shape of the epidemic curve suggested a common source outbreak. The first case–patient developed symptoms on May 31^st^, 2019, and it took two months for the first symptomatic case to appear. The spread peaked after 11 days, and cases slowly came to an end 55 days from the first appearance of the first case-patient. It is possible that this slow spread was linked to the fact that vectors were not abundant during this time. Larviciding was performed to control the development of mosquitoes which breed in this water despite the fact that no *An*. *balabacensis* larvae were found in ponds or other standing water. *Anopheles barbirostris* larvae were found in stagnant water flowing from a river near one of the positive case residencies. However, there is no report implicating *A*. *barbirostris* as a malaria vector in Malaysia. The infectiousness of *P*. *malariae* for *Anopheles* mosquitoes in malaria-endemic areas remains unclear and merits further investigation [17].

All families surveyed in the village during this investigation reported the use of ITNs. This may be one of the reasons that few children under five were infected during the outbreak. Although there were some differences in age distribution between symptomatic and asymptomatic cases, adults were most at risk followed by 5–18 year-olds. As there is no electricity in the village, families spend their time outside engaged in activities such as storytelling, eating together, playing games and web-surfing, and only go inside the house to sleep. This tendency to be outside during peak mosquito-biting time might contribute to the risk of infection.

A similar study from Indonesia also found that older people were more likely to be infected with *P*. *malariae* [16] than younger people. However, studies from Kenya and Tanzania have shown that *P*. *malariae* infection in children exclusively [17,18]. This discrepancy between the epidemiology of *P*. *malariae* in Africa and Asia may be due to multiple factors including differences in host and parasite genetics, cross-immunity between malaria parasite species, and host behavioral differences.

We observed that engagement in the logging industry is associated with increased *P*. *malariae* infection, further reinforcing the assumption that outdoor biting and proximity to forested areas contributes to infection risk. We assume that such individuals are infected whilst at work in the forest, but it is also possible that they are infected when returning from work late in the evening. This warrants the distribution of personal anti-mosquito repellents to villagers, as their activities such as farming, hunting and logging mainly occur outdoors. Insect repellents are an additional self-protection tool for avoidance of mosquito bites. These could complement other preventive measures such as IRS and the distribution of LLINs. The distributed repellent has a long lasting effect for up to eight hours.

Logging can drastically alter the forest landscape. Environmental modification through deforestation is an important driver of malaria transmission in Sabah [2–4], and the Brazilian Amazon [19]. In Vietnam it has been shown that working in the forest is a significant risk factor for malaria, which is further increased by staying in the forest overnight [20]. The same study found that lower socio-economic status and male gender were also found to pose an increased risk of infection with *P*. *malariae*, which is in agreement with the present study. Men are more likely than women to spend time outside houses during the evening, and will occasionally sleep outdoors, both of which increase the risk of infection.

Notably, we found that over 60% of the infections identified during this outbreak were asymptomatic. Asymptomatic malaria poses a challenge to malaria control and elimination programs, as it makes identification of parasite carriers difficult [21]. It is becoming apparent that *P*. *malariae* is more common than previously thought, and asymptomatic carriage of this parasite might be commonplace. This is due mainly to the increased sensitivity of malaria parasite detection methods, including PCR and LAMP technologies [22]. Surveys conducted by rapid diagnostic tests and microscopy may often underestimate the prevalence of *P*. *malariae* because parasitaemia is typically below the threshold of detection [7,10]. Several studies have confirmed that the blood stage of *P*. *malariae* can persist for extremely long periods, asymptomatically [7,8,10].

Despite the high attack rate in the present report, there were no cases of anemia or nephritis, which are the most serious clinical manifestation of *P*. *malariae* [7–9]. Although it has been reported that *P*. *malariae* may be intrinsically less susceptible to artemisinin than *P*. *falciparum* [11,12], all patients in the current outbreak responded to treatment indicating that the strain was not resistant to the anti-malarials used.

Adequate-quality housing reduces the opportunities for entry of vectors and can radically reduce the human biting rate of malaria-transmitting mosquitoes [23]. We found several incomplete housing structures in Sonsogon Paliu which might be a significant risk factor for malaria infection in their residents. Improving house design and location should be considered as a potential malaria control method in villages [24].

In conclusion, we describe an outbreak of *P*. *malariae* in the village of Sonsogon Paliu in Malaysian Borneo. The index case was an immigrant worker from Sulawesi, Indonesia who was working for a logging company in the area. We identified a cluster of sub-microscopic, asymptomatic infections, and analyzed the risk factors associated with parasite carriage. We suggest that district health offices should collaborate and co-ordinate with the administration section of commercial logging companies in this region in order to consolidate the effective implementation of anti-malaria interventions among workers. Methods for interrupting the exposure of workers to malaria vectors inside the forest need to be formulated in order to prevent further outbreaks.

## Supporting information

S1 Table Questionnaire used during the *Plasmodium malariae* outbreak in Sonsogon Paliu village. (DOCX)

S1 Fig Sonsogon Paliu village could be accessed only by using 4×4 vehicle. Photographer: Nurul Athirah Naserrudin.
(DOCX)

S2 Fig Piles of timber along the roadside heading towards Sonsogon Paliu village. Photographer: Nurul Athirah Naserrudin.
(DOCX)

S3 Fig The road heading towards Sonsogon Paliu village. Photographer: Nurul Athirah Naserrudin.
(DOCX)
