# Supplementary material for: High incidence of asymptomatic cases during an outbreak of Plasmodium malariae in a remote village of Malaysian Borneo
Source: PLoS Negl Trop Dis. 2021 Jun 3;15(6):e0009450. doi: 10.1371/journal.pntd.0009450 (PMC8205135; doi:10.1371/journal.pntd.0009450)
Supplement: S1 Table — (DOCX) [file pntd.0009450.s001.docx]

Questionnaire for the case-control study during the *P. malariae* outbreak in Songsogon Paliu

1. Demographic information
   1. Age
   2. Are you married?
   3. How many children do you have?
   4. How many people staying together in your house?
   5. Work – farming? logging company worker?
   6. Toilet – is your toilet/bathroom inside/outside the house?
   7. Are there ponds/ stagnant water near home?
   8. Home condition – complete/not complete / bamboo house?
   9. Education level?
2. Lifestyle
   1. What do you do at night/late evening?
   2. Do you often stay outside of home at night/late evening?
   3. Do you go hunting?
   4. Do u go fishing/river activity?
   5. Where do you go to buy food stocks?
   6. Where do you go to get the telephone signal?
3. Preventive measures against malaria mosquito bites
   1. Do you used bed nets at night?
   2. How many bed nets do you have at home?
   3. Do you use insect repellent?
   4. Do you wear long shirt/long trousers during work/at night during activity?
   5. When was the last time your house sprayed by the healthcare team with insecticide?
4. Comorbid conditions/ Medical history
   1. Have you had malaria infection previously?
   2. Have you any medical illness?
   3. Are you on any medications from the hospital/clinic?
   4. Are you on any medications that you buy from pharmacy/ herbal shop?
   5. Have you had any blood transfusion previously?
5. Do you use any traditional medicine/plants when you feel sick/fever?
6. Symptoms
   1. Do you have any fever for the past 1 month?
   2. Do you have any chills/rigors/body weakness in the past 1 month?
7. Travel history

Have you travelled out of the village during the past 1-2 months?

If the patient has malaria – we will ask his travelling history for the past 1 months (by day)

1. Knowledge regarding malaria
   1. Have you heard of malaria before?
   2. What are the signs/symptoms of malaria?
2. Do you see any monkey near your house?
3. Recently did any foreigner come to your village?
