# Supplementary figures and images for: High incidence of asymptomatic cases during an outbreak of Plasmodium malariae in a remote village of Malaysian Borneo
Source: PLoS Negl Trop Dis. 2021 Jun 3;15(6):e0009450. doi: 10.1371/journal.pntd.0009450 (PMC8205135; doi:10.1371/journal.pntd.0009450)

S1 Fig.


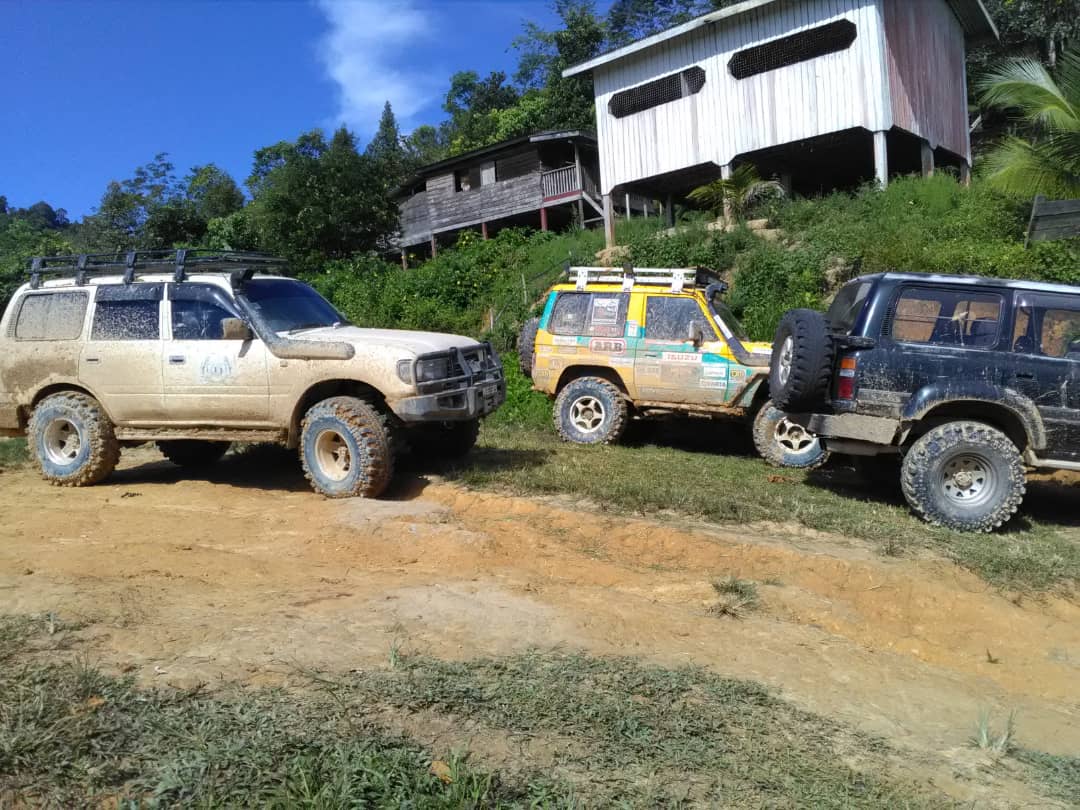

Supplement: S1 Fig — Photographer: Nurul Athirah Naserrudin. (DOCX) [file pntd.0009450.s002.docx]

S2 Fig.

~~
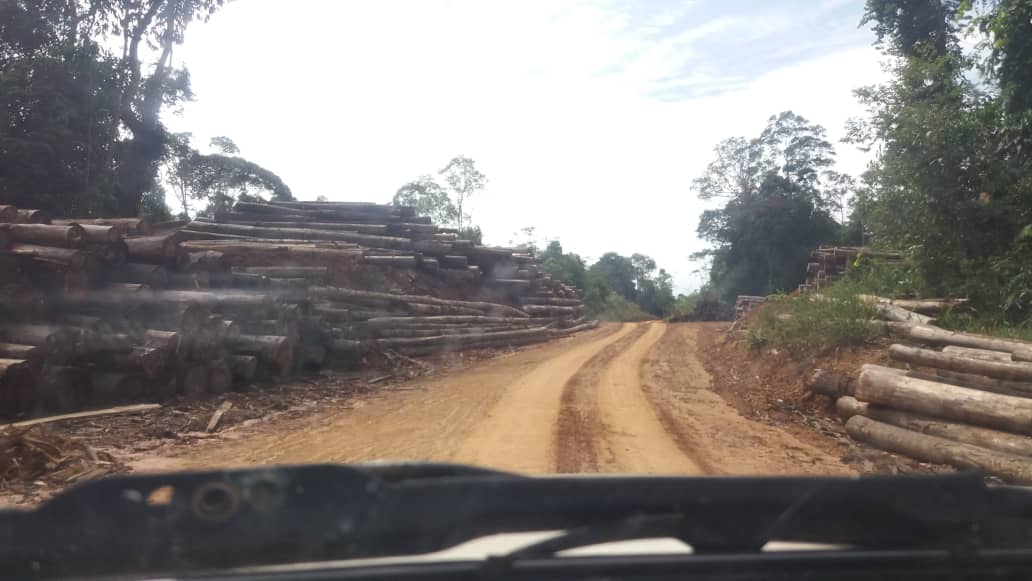
~~

Supplement: S2 Fig — Photographer: Nurul Athirah Naserrudin. (DOCX) [file pntd.0009450.s003.docx]

S3 Fig.


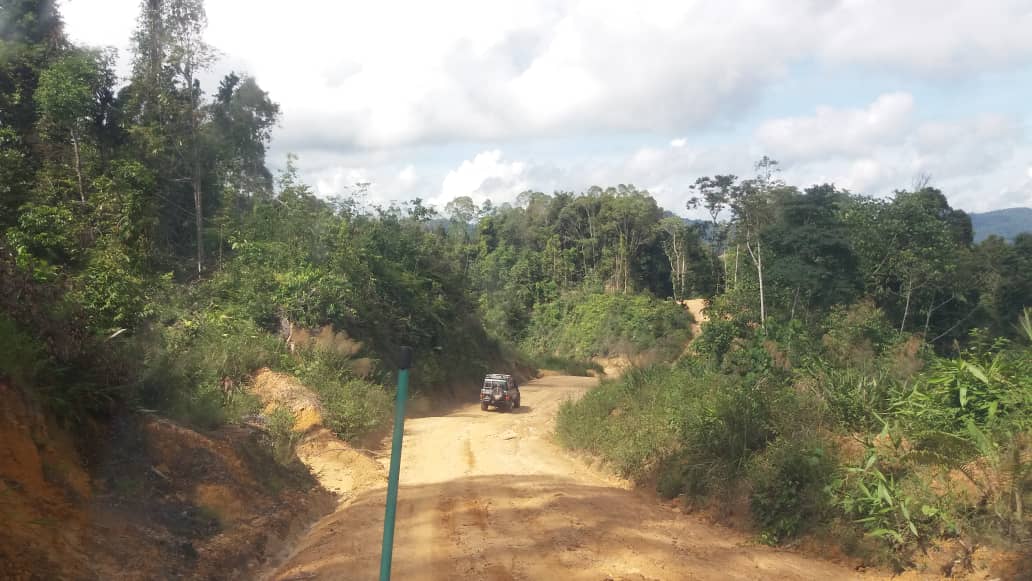

Supplement: S3 Fig — Photographer: Nurul Athirah Naserrudin. (DOCX) [file pntd.0009450.s004.docx]
